# Descriptive analysis of diseases, non-battle injuries and climate among deployed Swedish military personnel

**DOI:** 10.1136/military-2024-002685

**Published:** 2024-08-25

**Authors:** Matilda Saleby, L Ahlinder, M Schüler, F Taube

**Affiliations:** 1 Anesthesiology and Intensive Care, Sahlgrenska University Hospital, Gothenburg, Sweden; 2 Swedish Defence Research Agency, Umea, Sweden; 3 Strategy Department/Research and Development, Swedish Defence University, Stockholm, Sweden; 4 Research Centre for Disaster Medicine, Institute for Clinical Sciences, Sahlgrenska Academy, Gothenburg, Sweden; 5 School of Public Health and Community Medicine, Sahlgrenska Academy, Gothenburg, Sweden

**Keywords:** PRIMARY CARE, STATISTICS & RESEARCH METHODS, ACCIDENT & EMERGENCY MEDICINE, HEALTH SERVICES ADMINISTRATION & MANAGEMENT, Health informatics, Information management

## Abstract

**ABSTRACT:**

**Introduction:**

Historically, diseases and non-battle injuries (DNBI) typically stand for 70%‒95% of all medical events during military missions. There is, however, no comprehensive compilation of medical statistics for Swedish soldiers during deployment.

**Method:**

During United Nations Multidimensional Integrated Stabilization Mission in Mali, climate data and medical outpatient health surveillance data were compiled for Swedish soldiers deployed to Timbuctoo, between 2015 and 2019. Correlations between climate data and medical outpatient health surveillance data were analysed.

**Results:**

Battle injuries accounted for 0.4% of the visits to healthcare, while diseases accounted for 53.6%, and non-battle injuries for 46%, the majority being musculoskeletal injuries. The combination of high temperature, humidity, sun radiation and good visibility, during summer rotation weeks, caused more events of injuries and heat stress than any other period.

**Conclusion:**

Musculoskeletal injuries were the major cause for visits to the Swedish camp hospital. Injuries and heat stress increased during periods of high temperature, humidity, sun radiation and good visibility. Lack of medical data, i.e. unknown number of unique patients seeking healthcare, cause codes not always connected to a primary diagnosis, and revisits not being connected to a diagnose, complicated interpretation of health risk factors.

**WHAT IS ALREADY KNOWN ON THIS TOPIC**

- Diseases and non-battle injury during deployments are much more common than battle injury (BI).

**WHAT THIS STUDY ADDS**

- Documentation of outpatient care in a Swedish camp hospital during a deployment.
- A foundation for better understanding of health conditions in outpatient settings.

**HOW THIS STUDY MIGHT AFFECT RESEARCH, PRACTICE OR POLICY**

- Optimisation of medical surveillance and of training and educational programmes.

## Background

 In modern warfare, diseases and non-battle injuries (DNBI) accounts for 70%‒95% of the medical evacuations/hospitalisations among soldiers.^1^,^6^ This can be exemplified by data from the Korean War (1950‒1953) and Gulf War (1990‒1991), when medical evacuations due to DNBIs went from almost 600 per 1000 person-years to less than 100 per 1000 person-years. With an improved Deployment Health Surveillance Capability, evacuations/hospitalisation due to DNBI decreased further during the wars in Iraq and Afghanistan.^7^,^11^ Historically, musculoskeletal/orthopaedic diagnoses caused by pre-existing disorders or by overuse accounts for a large part of medical evacuations and lost duty days.^12^,^16^

The Swedish Armed Forces participated in United Nations Multidimensional Integrated Stabilization Mission in Mali (MINUSMA) established in April 2013. The harsh environmental conditions in Mali prompted a high awareness of health-related issues and earlier studies on Swedish personnel stationed in desert environments have highlighted the relationship between chronical respiratory symptoms and time spent in desert environment.^17^

The aim of this study is to present morbidity in the Swedish contingency in Mali during the period from May 2015 to November 2019 (223 weeks), together with correlates to climate data.

## Methods

### Population data

In total 2 500 persons rotated to Mali during the study period, with 250 militaries in each rotation, that is, ‘winter rotation’ during November/December and ‘summer rotation’ during May/June. Each rotation brought a medical capacity including physicians, nurses, psychologist, physiotherapist and unlicensed healthcare workers.

### Medical record data

Visits to the Swedish contingency hospital care at Camp Nobel in Timbuctoo were compiled on a weekly basis, together with diagnose code categories according to International Classification of Diseases, 10th revision (ICD-10). Rates for compiled categories were expressed in events per 1000 person-years or 100 person months.

The term ‘NBI’ includes the categories injuries (S and T diagnostic codes) and musculoskeletal (M diagnostic codes).^5 13 18 19^ Revisits, hospitalisations, evacuations, cause codes and response codes could not be matched with their corresponding primary diagnosis in the data set. Apart from the cause code ‘war operation unspecified’ (Y 36.9) categorised as battle injuries (BI), cause codes and response codes were, therefore, excluded from the calculations.

### Climate data

Climate data, including Wet Bulb Globe Temperature (WBGT), a measure of the exposure to environmental heat on humans that considers temperature, humidity, wind speed, sun angle and cloud cover,^20^ and hourly measured values for visibility (km) and relative humidity (%) was compiled together with medical record data. Data were either transformed into weekly minimums and maximums (visibility and relative humidity) or means (WBGT and temperature). For days that lacked data on WBGT, mean values were estimated based on the days before.

### Data preparation

#### Scale variables

Medical data were summarised into 13 categories based on the ICD-10 diagnose categories (online supplemental file 1). For climate data, visibility, being a proxy for particulates in the air, was chosen as a scale variable due to its impact on the personnel’s ability to work, regardless of WBGT.

#### Categorical variables

To calculate differences between measured WBGT values, WBGT was transformed into a normally distributed categorical variable (WBGT*), resulting in six temperature categories: 19.33‒23.05, 23.06‒27.00, 27.01‒30.95, 30.96‒33.20, 33.21‒34.43 and 34.44‒36.26 (C°). The variable "rotation", which differentiates between summer and winter rotation, was added to the data set to incorporate more contextual information into the statistical analysis. Each week was categorised as either 0 (normal week), 1 (winter rotation week) or 2 (summer rotation week).

### Data analysis

WBGT* was analysed for skewness and kurtosis. Post hoc test was used to analyse differences between WBGT* groups. Bivariate correlation analysis was conducted on the 13 ICD-10 diagnose categories and WBGT*. Three separate one-way ANOVA (Analysis of Variance) calculations was conducted: WBGT* and Heat stress, Rotation and Heat stress and WBGT* and Respiratory, respectively. A univariate analysis was conducted on heat stress and (WBGT*×Rotation) with visibility as a WLS-weight (weighted least squares). F-values was used to calculate the between group variation and within group variation. A large F-value is interpreted as a statistically significant difference between groups. IBM SPSS, V.29.0.1.0 was used in all calculations.

## Results

### Morbidity

The study period, corresponding to 1 250 person-years, generated 5 852 diagnostic codes. Given that only 62 patients were hospitalised and seven medically evacuated out of the 5774 clinical visits, this was primary and outpatient study. DNBI was found to be 39.0 events per 100 person months, while NBI and BI generated 18.1 and 0.2 events per 100-person month, respectively (table 1).

**Table 1:** Rates for compiled ICD-10 categories expressed in events per 1000 person-years and 100 person months, respectively

Categories	Events/1000 person-years	Events/100 person months
DNBI	4681.6	39.0
NBI (musculoskeletal+injuries)	2168.0	18.1
Musculoskeletal	1234.4	10.3
Injuries	933.6	7.8
Skin	608.8	5.1
Respiratory	528.8	4.4
Ill-defined	422.4	3.5
Infectious	354.4	3.0
Digestive	198.4	1.7
Behavioural	139.2	1.2
Eye	116.0	1.0
Ear	74.4	0.6
Genitourinary	43.2	0.4
Other	22.4	0.2
BI	18.4	0.2
Circulatory	4.0	0.0
Nervous	1.6	0.0

Calculations made from a total of 1250 person-years.

BI, battle injury; DNBI, diseases and non-battle injuries; ICD-10, International Classification of Diseases, 10th revision.

**Figure 1:**
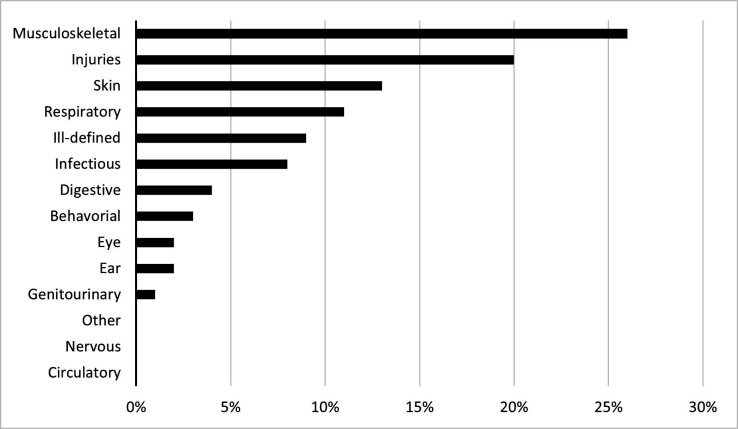
Distribution of categories in per cent of the total number of diagnose codes (n=5852) reported during medical visits of the Swedish military between 2015 and 2019 in Mali.

The four leading diagnostic categories were ‘musculoskeletal’ (26%), ‘injuries’ (20%), ‘skin’ (13%) and ‘respiratory’ (11%), accounting for 70% of the total reported diagnosis (figure 1). The top two diagnoses in the NBI category were ‘myalgia’ (15%) and ‘low back pain’ (13%), contributing to more than 25% of all NBI. According to physiotherapists at site during 2019, commonly exposed body parts were neck/cervical spine (26%), lumbar spine/pelvis (17%), shoulder/arm (15%) and lower leg/foot (15%).

Four types of cause codes were reported, including 12 reports of ‘person injured in unspecified vehicle accident’ (V89.9), 107 reports of ‘striking against or struck by sports equipment’ (W21), 18 reports of ‘exposure to noise’ (W42) and 17 reports of ‘war operations unspecified’ (Y36.9). Three types of response codes were reported, including 23 reports of ‘issue of medical certificate’ (Z02.7), 5 reports of ‘need for immunisation against unspecified infectious diseases’ (Z26.9) and 137 reports of ‘observation for disease’ (Z03.9).

### Correlation of medical record and climate data

The categorial variable WBGT* produced acceptable values for skewness (−0.03) and kurtosis (−0.98). The bivariate correlations between the category groups, Heat stress and WBGT* produced several small correlations below 0.36. The variables heat stress (0.18) and respiratory (0.31) produced small but statistically significant correlations with WBGT*. There was a statistically significant correlation between visibility and injuries (−0.20), indicating more events of injuries when visibility is good.

ANOVA test between WBGT* and heat stress (table 2) was statistically significant and the post hoc analysis produced several significant differences between the category 33.21–34.43 (a) and the categories in the range 19.33–33.20 (b). Events of heat stress recorded in the category 33.21–34.43 (a) were significantly higher than in any other category. ANOVA test between WBGT* and respiratory was statistically significant. Post hoc analysis produced several significant differences between the category 34.44–36.26 (a) and the categories in the range 19.33–30.95 (b), and between the category 33.21–34.43 (c) and the categories in the range 19.33–30.95 (d). Events of respiratory diagnoses recorded in the highest WBGT category 34.44–36.26 (a) was significantly lower than in the other WBGT categories.

**Table 2:** One-way ANOVA between WBGT * and Heat stress and WBGT* and Respiratory

WBGT*	Heat stress				Respiratory			
	df(F)	n	M	SD	df(F)	n	M	SD
19.33‒23.05	206 (2.70)^*^	19	0.00^**b^	0.00	206 (4.20)**	19	3.47*^bd^	2.76
23.06‒27.00		38	0.05**^b^	0.32		38	3.79**^bd^	2.06
27.01‒30.95		46	0.02**^b^	0.15		46	3.76**^bd^	2.50
30.96‒33.20		42	0.10*^b^	0.37		42	2.81	2.02
33.21‒34.43		43	0.28^a^	0.59		43	2.16^c^	2.29
34.44‒36.26		19	0.16	0.50		19	2.00^a^	1.53

*p<0.05, **p<0.01, statistically significant differences between (a – b and c – d). ‘n’ = number of weeks in each category. ‘M’ = average number of events of heat stress and respiratory symptoms respectively in each category.

ANOVA = Analysis of Variance

WBGT, Wet Bulb Globe Temperature.

ANOVA test between rotation and heat stress (table 3) was statistically significant. Post hoc analysis produced two significant differences between the categories summer rotation (a) and no rotation and winter rotation (b). Events of heat stress during summer rotation were significantly higher.

**Table 3:** One-way ANOVA between Rotation and Heat stress

Rotation	df(F)	n	M	SD
No rotation	206 (4.14)*	163	0.09^b^	0.34
Winter rotation		17	0.00^b^	0.00
Summer rotation		27	0.30^a^	0.67

*p<0.05, **p<0.01, statistically significant differences between (a) and (b). ‘n’ = number of weeks in each category, ‘M’ = average number of events of heat stress in each category.

ANOVA = Analysis of Variance

The univariate analysis between heat stress and (WBGT*×Rotation) was not statistically significant. However, by adding visibility to the univariate analysis as a WLS-weight, the analysis became statistically significant (table 4).

**Table 4:** Tests of between-subjects: Heat stress and (WBGT* × Rotation)

Source	df	M. square	F
WBGT*	5	4.09	2.75*
Rotation	2	4.56	3.06*
WBGT* x Rotation	5	4.04	2.71*

*p<0.05.

**p<0.01.

WBGT, Wet Bulb Globe Temperature.

**Figure 2:**
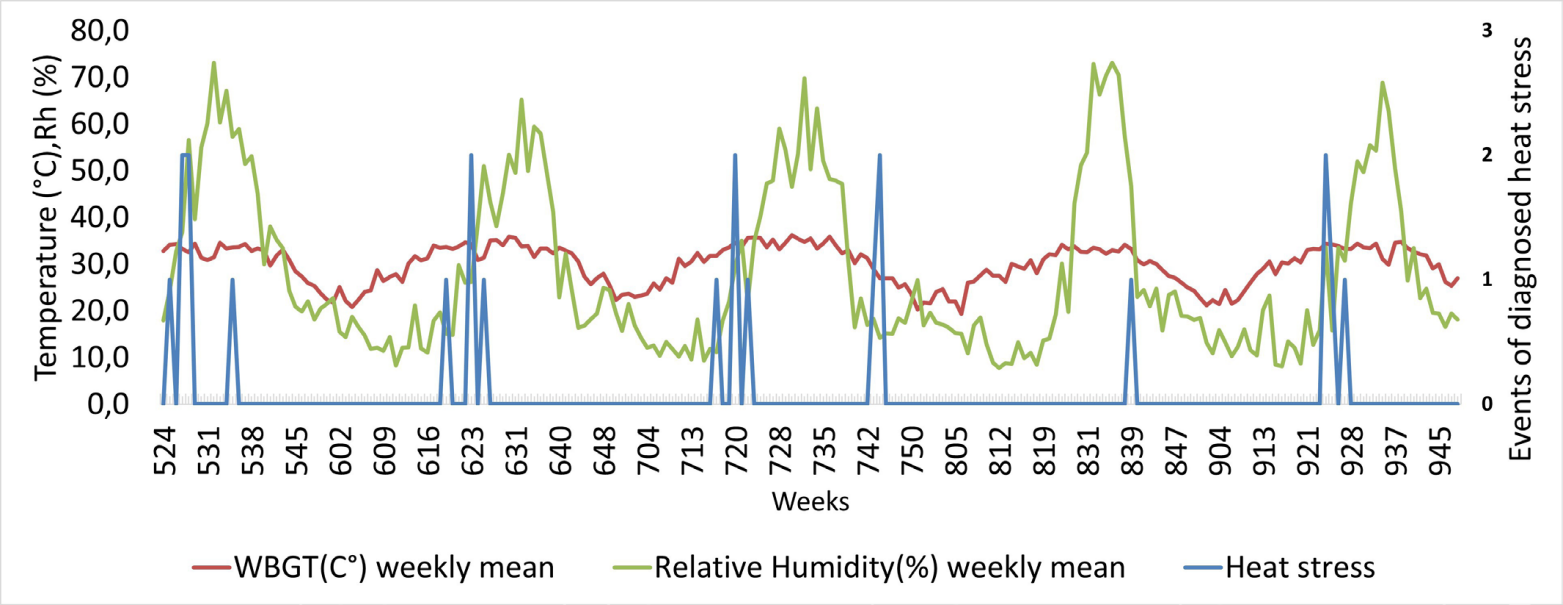
Events of heat stress (T67.5) visualised together with WBGT and relative humidity, between 2015 and 2019. The x-axis represents a total of 223 weeks, where week 945 corresponds to the year (9 for 2019) and specific week that year (number 45, ie, in October), while week 524 corresponds to the year 2015 and specific week 24, ie, in June). WBGT, Wet Bulb Globe Temperature.

Figure 2 illustrates the correlation of climate data with events of heat stress (n=22). The univariate analysis shows that high WBGT* during summer rotation weeks when the visibility is good, generated more events of heat stress than high WBGT* values do during any other weeks.

## Discussion

This study adds important documentation of outpatient care during a deployment. We found 39 events of DNBI per 100 person months, which is in line with earlier studies using outpatient health surveillance data.^17 21 22^ Of these, 18 events per 100 person months are due to NBI, ie, 2–4 times higher than reported in earlier studies.^15 6 8^,^10 21^ Many serious injuries are treated on an outpatient basis, as well as conditions considered to be of moderate severity, such as back pain, knee pain and myalgia, despite their potential of being severely debilitating and disabling.

Historically, physiotherapy treatment accounts for a large part of the visits,^19^ due to symptoms from lower back and shoulder, knee and neck and lumbar spine.^23^ Poor ergonomic conditions and over-use from sport activities contribute to these injuries. Especially sport activities have been reported to be a major cause for hospitalisation^5 22 24^ and in the present study, more than 100 cause codes for ‘sport-activity’ were reported. Injury-related musculoskeletal conditions are usually due to repetitive or cumulative microtrauma, which suggests that conditions such as myalgia, low back pain, joint pain, and enthesopathies can be prevented by intervening in the repetitive cycle.

Dermatological disorders typically account for 10% to 15% of the outpatient visits,^18 22^ whereas in studies based on hospitalisation and evacuation data, only 1%–1.5% are due to skin disorders.^6 24^ A probable reason for this is that skin disorders generally are not critical.^12^

Eleven per cent of all visits were due to respiratory diseases, of which acute upper respiratory infection was pronounced during rotation. In McKee *et al*, ‘respiratory’ was the second largest category, in which ‘acute upper respiratory infection’ dominated.^19^ Cox *et al* found a higher frequency of gastroenteritis during rotation.^22^ In the present study, 8% of all diagnoses were due to infections, but the reported cases did not increase during rotation.

An increase of heat stress and a decrease in respiratory symptoms was found with increasing WBGT*. In addition, there was a statistically significant correlation between visibility and injuries, in that more injuries were reported during episodes of good visibility. These results indicate that visibility do constrain military activity, and from an activity theoretical perspective,^25^ visibility can be seen as an implicit rule influencing and constraining^26^ how military personal solve military problems. Finally, the combination of high WBGT* and good visibility during summer rotation weeks, caused more events of injuries and heat stress than any other period.

### Limitations

In the present study, medical data for 28 weeks were missing due to lack of routines for reporting. Problems related to imprecisely measured populations at risk are common in studies of disease frequency, so also in the present study, where the actual number of unique patients seeking healthcare was not available. Since revisits were not connected to a diagnose, one unique diagnosis could be counted more than once. Finally, treatment executed by a medical unit following a smaller troop on a task away from the camp might not have been compiled in the ICD-10 reports. The strengths of the present study are long duration time, diagnoses made by medical care providers, medical data tabulated using ICD-10 codes and the simultaneous collection of climate data.

## Conclusions

NBI were the leading reason for deployed Swedish soldiers to seek medical care, partly because of poor ergonomic working environment and risky behaviour during exercise or sport activity. The combination of high WBGT* and good visibility during summer rotation weeks, caused more events of injuries and heat stress than any other period.

Low medical data quality complicated the interpretation of health risk factors for the Swedish contingency during MINUSMA. Future studies on outpatient settings could be significantly improved by a centralised surveillance, better documentation of denominators and by connecting cause codes, hospitalisations, evacuations and revisits to its specific ICD code.

The present study provides a foundation for better understanding of the most common health conditions affecting soldier health in outpatient settings and for setting priorities for prevention and treatment of the most common conditions affecting solider health. Tracking climate and seasonal data may help predict some health conditions, such as heat-related diseases.

## Supplementary material

10.1136/military-2024-002685 online supplemental file 1

## Data Availability

Data are available upon reasonable request.
