# Supplementary material for: Descriptive analysis of diseases, non-battle injuries and climate among deployed Swedish military personnel
Source: BMJ Mil Health. 2024 Aug 25;171(e1):e002685. doi: 10.1136/military-2024-002685 (PMC12772538; doi:10.1136/military-2024-002685)
Supplement: online supplemental file 1 [file military-171-e1-s001.pdf]

Supplement 1: Groupings of ICD-10 diagnosis codes

Infectious:
A09 Other gastroenteritis and colitis of infectious and unspecified origin
B34.9 Viral infection, unspecified
B54 Unspecified malaria
B55.1 Cutaneous leishmaniasis
B65.9 Schistosomiasis, unspecified
B99 Other and unspecified infectious diseases
B26.9 Mumps without complication
B35.3 Tinea pedis
Behavioral health:
F32.9 Depressive episode, unspecified
F43.9 Reaction to severe stress, unspecified
F51.9 Nonorganic sleep disorder, unspecified
Nervous system:
G56.0 Carpal tunnel syndrome
G44.2 Tension-type headache
Diseases of the eye and the adnexa:
H10.9 Conjunctivitis, unspecified
H57.9 Disorder of eye and adnexa, unspecified
H16.0 Corneal ulcer
Diseases of the ear and mastoid process:
H60.9 Otitis externa, unspecified
H91.9 Hearing loss, unspecified
H61.2 Impacted cerumen
H66.0 Acute suppurative otitis media
H93.1 Tinnitus
Circulatory:
I80.3 Phlebitis and thrombophlebitis of lower extremities, unspecified
I49.9 Cardiac arrhythmia, unspecified
Rheumatic fever without mention of heart involvement
Respiratory:
J06.9 Acute upper respiratory infection, unspecified
J22 Unspecified acute lower respiratory infection
J98.9 Respiratory disorder, unspecified
J45.9 Asthma, unspecified
J40 Bronchitis, not specified as acute or chronic
J02.9 Acute pharyngitis, unspecified
J11.1 Influenza with other respiratory manifestations, virus not identified
J03.9 Acute tonsillitis
J30.0 Vasomotor rhinitis
R05 Cough
Digestive:
K30 Functional dyspepsia
K59.0 Constipation
K59.1 Functional diarrhoea
K12 Recurrent oral aphthae

K05.0 Acute gingivitis
K02.9 Dental caries, unspecified
K64.9 Hemorrhoids, unspecified
Skin:
L03.0 Cellulitis of finger and toe
L08.9 Local infection of skin and subcutaneous tissue, unspecified
L30.9 Dermatitis, unspecified
R23.8 Other and unspecified skin changes
L60.0 Ingrowing nail
L72.0 Epidermal cyst
Musculoskeletal:
M25.5 Pain in joint
M54.2 Cervicalgia
M54.5 Low back pain
M75.1 Rotator cuff syndrome
M77.9 Enthesopathy, unspecified
M79.1 Myalgia
M54.8 Other dorsalgia
M77.1 Lateral epicondylitis
M72.2 Plantar fascial fibromatosis
M76.5 Patellar tendinitis
M21.3 Wrist or foot drop
M54.4 Lumbago with sciatica
M65.3 Trigger finger
M10.9 Gout, unspecified
M67.4 Ganglion
Genitourinary:
N30.0 Acute cystitis
N43.3 Hydrocele, unspecified
N76.8 Other specified inflammation of vagina and vulva
N61 Inflammatory disorders of breast
R39.1 Other difficulties with micturition
A64 Unspecified sexually transmitted disease
A74.9 Chlamydial infection, unspecified
Ill-defined conditions:
R68.8 Other specified general symptoms and signs
R00.2 Palpitations
R42 Dizziness and giddiness
R50.9 Fever, unspecified
R51 Headache
R55 Syncope and collapse
R59.9 Enlarged lymph nodes, unspecified
R04.0 Epistaxis
R10.4 Other and unspecified abdominal pain
R11 Nausea and vomiting
R13 Dysphagia
Ear, nose and throat diseases, unspecified
Injuries:

S06.0 Concussion
S09.9 Unspecified injury of head
S02.5 Fracture of tooth
S29.9 Unspecified injury of thorax
S39.9 Unspecified injury of abdomen, lower back and pelvis
S49.9 Unspecified injury of shoulder and upper arm
S59.9 Unspecified injury of forearm
S69.9 Unspecified injury of wrist and hand
S79.9 Unspecified injury of hip and thigh
S89.9 Unspecified injury of knee and lower leg
S99.9 Unspecified injury of ankle and foot
T30.0 Burn of unspecified body region, unspecified degree (Burn NOS)
T15.9 Foreign body on external eye, part unspecified
T14.1 Open wound of unspecified body region (animal bite)
T35.7 Frostbite NOS
T63.9 Toxic effect of contact with unspecified venomous animal
T67.5 Heat stress, unspecified
H83.3 Noise effects on inner ear
Other:
T78.4 Allergy, unspecified
Pregnancy confirmed
Wisdom tooth
Straighten irregular teeth
Tootache
